# Solid basal adenoid cystic carcinoma of the breast: A case report and literature review

**DOI:** 10.1097/MD.0000000000037010

**Published:** 2024-01-19

**Authors:** Wen Bin Gou, Yong Qiang Yang, Bei Wen Song, Pei He

**Affiliations:** a Department of Pathology, People’s Hospital of Wanning, Wanning, Hainan, China; b Department of Endoscopy, People’s Hospital of Wanning, Wanning, Hainan, China; c Department of Clinical laboratory, Xinjiang Production and Construction Corps Sixth Division Hospital, Wujiaqu, Xinjiang, China.

**Keywords:** adenoid cystic carcinoma, breast, diagnosis, prognosis, treatment

## Abstract

**Rationale:**

Adenoid cystic carcinoma (AdCC) is a rare malignancy of the breast with a low Ki-67 index and good prognosis. Owing to the rarity of breast AdCC, the misdiagnosis rate is as high as 50%, and there is no consensus or recognized guidelines for the treatment of this disease. Therefore, it is necessary to conduct a detailed clinical and pathological analysis in combination with a literature review to improve our understanding, diagnosis, and treatment of the disease.

**Methods:**

A 68-year-old woman sought medical attention due to a recently increasing mass in the breast. The left breast mass was 1.3 cm × 1 cm in size. We analyzed the morphology, immunohistochemistry, and molecular characteristics of the tumor removed by surgery, and reviewed relevant literature.

**Diagnoses:**

Solid basal AdCC of the breast.

**Interventions:**

We performed biopsy, immunohistochemistry and molecular testing on surgical resection specimens.

**Outcomes:**

Combining morphological and immunohistochemical features, it is consistent with solid basal AdCC of the breast, and Fish detected MYB gene break.

**Lessons:**

Due to the high misdiagnosis rate of AdCC, accurate histopathological diagnosis is particularly important. At present, breast conserving surgery and local tumor resection are mainly used for the treatment of breast AdCC, and postoperative adjuvant radiotherapy is feasible.

## 1. Introduction

Adenoid cystic carcinoma (AdCC) is a rare malignant tumor of the breast, accounting for 0.1% to 3.5% of all breast tumors. Breast AdCC is a specific subtype of triple-negative breast cancer (TNBC) and its histological presentation is different from that of invasive ductal carcinoma.^[1–5]^ However, it has a better prognosis than the other subtypes, with 5-, 10- and 15-year survival rates reaching 98.1%, 94.9%, and 91.4%, respectively.^[6]^ The fifth edition of the WHO guide classifies AdCC into 3 categories: classic AdCC (C-AdCC), solid basaloid AdCC (SB-AdCC), and AdCC with high-grade transformation.^[7]^ SB-AdCC is usually characterized by solid nests composed of basal-like cells with obvious atypia, frequent mitotic figures, and visible necrosis. Due to its rarity, it is urgently needed to report new cases of SB-ADCC in order to improve the level of diagnosis and treatment of the disease. Here we report a case of SB-AdCC of the breast with detailed clinical, histological, and immunohistochemical, including the FISH detection of MYB gene-break.

## 2. Case presentation

A 68-year-old woman sought medical attention due to a recently increasing mass in the breast. The lump was located in the areolar area of the left breast at 4 o’clock. It had a size of approximately 1.0 cm × 1.5 cm and was tough, with clear boundaries, good mobility, and no adhesion to the skin. Breast ultrasound: Focal ultrasound of the left breast, BI-RADS Class 4A. Gross: The left breast mass was 1.3 cm × 1 cm in size. The mass was grayish-white with unclear boundaries and was of medium quality.

**Figure 1.**
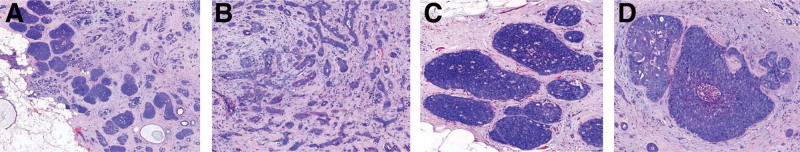
(A) The tumors showed solid/basal-like, cribriform, tubular, and beam-like growth. (B) The tumor cells resembled the shape of sieves, tubules, or beams, and exhibited true glandular cavities. (C/D) The tumor showed solid/basal-like growth with sebaceous adenoid differentiation and a pseudoglandular lumen.

Morphological characteristics of the tumor was composed of cells differentiated from glandular, myoepithelial/basal-like, and sebaceous glands, forming 4 types of structures: sieve, tubular, beam, and solid/basal-like (Figs. 1A and B). SB-AdCC accounted for approximately 35% of the tumor components (Figs. 1C and D), and nests of infiltrating solid/basal-like cell were observed in the fibrous stroma. The tumor cells were relatively and consistent with basal-like characteristics, little cytoplasm and medium to high nuclear level. The nuclei were elliptical or angular, with chromatin clumps, spots, or dense stained areas, and occasional nucleoli. There were more than 5 conspicuous mitotic images per 10 high-power fields. Both true and false glandular cavities were observed, whereby the true glandular cavities were composed of slightly larger surrounding eosinophilic cells, while the false glandular cavities contained mucoid or eosinophilic globules, and alternatively inflammatory necrotic lesions composed of basement membrane-like material surrounded by myoepithelial/basal-like cells with little cytoplasm and deeply stained nuclei.

**Figure 2.**
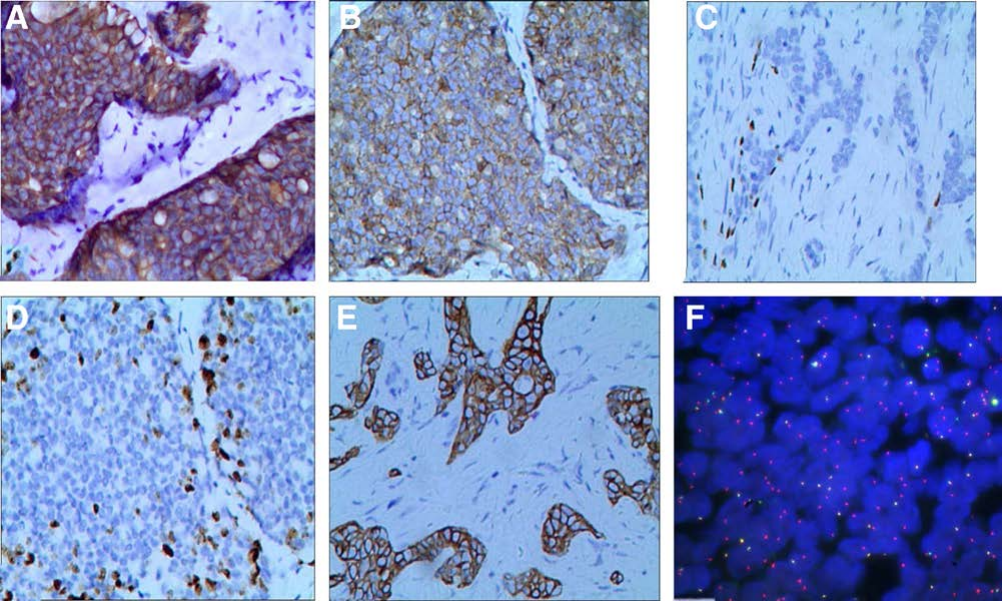
(A) The tumor cells showed diffuse CK7 positivity. (B) The tumor cells were diffusely positive for CD117. (C) Periglandular myoepithelial cells were P63 positive. (D) About 30% of the tumor cells in the hotspot were Ki67 positive. (E) The tumor cells were CK5/6 positive. (F) FISH results showed MYB gene-break.

The 2-step EnVision method was used to stain paraffin sections of tumor tissues (Figs. 2A–E), and the results are summarized as follows: CK7 (+), CD117 (+), calponin (+), SMA (+), focal (+), according to (+), CK5/6 (+), E-cadherin (+), P120 (+) membrane, EGFR (−) and ER (−), PR (−), Her-2(0), GATA-3(focal +), and Ki67 (hotspots with more than 30%).

FISH detection was performed on 4 μm thick paraffin sections using a Guangzhou LBP Mediciane MYB (6q23) gene-break probe. FISH detection results: MYB fracture probe (GSP MYB red fracture probe and GSP MYB green fracture probe). A total of 100 cells were counted, whereby 1R1G1Faccounted for 5%, 1R1G accounted for 1%, 1R1F accounted for 48%, 2R1F accounted for 20%, and nR1F accounted for 8% (n ≥ 3). The test results were positive, indicating MYB gene break (Fig. 2F).

## 3. Discussion

AdCC mainly occurs in the salivary glands, lungs, mammary glands, skin, and external ear canal, as well as in the digestive tract and uterus.^[8,9]^ Breast AdCC is more common in women aged 50 to 60 years and most cases present with a palpable mass. It is mostly located in the outer upper quadrant, under the areola, or in the central region, and in a few cases, it can involve the skin. Tumors are mostly solitary, and multiple tumors are rare.^[10,11]^ The tumor in this case was located in the areolar area of the left breast at 4 o’clock, which is consistent with literature reports. Radiographically, breast AdCC often presents as a well-defined lobulated mass with unclear or blurred borders that may be accompanied by microscopic calcification.

The histological presentation of breast AdCC is similar to that of salivary gland AdCC, and the tumor usually consists of adenoid-differentiated epithelial and myoepithelial/basal cell populations in sieve, tubular, trabecular, and solid/basal-like arrangements, with a single tumor generally containing a mixture of these structures. In addition, differentiation of sebaceous cells can also be seen.^[10]^ Basal-like cells usually express CK5, CK5/6, CK14, and CK17, while myoepithelial cells express p63, actin, calcium-binding protein, and S-100. Luminal cells expressed CK7, EMA, CEA, and CD117.^[12–14]^ Both cell types were negative for ER, PR and Her-2.

The pathological grading of ACC is controversial, but when the tumor is mainly tubular, with sieve and beam structures, it can be classified as grade I. A tumor with solid/basal-like composition < 30% is classified as histological grade II, while a tumor with solid/basal-like composition ≥ 30% is classified as histological grade III. A higher percentage of solid/basal-like components is associated with a worse prognosis.^[15]^

Recently, some scholars have reported rare gene changes in AdCC,^[13,16]^ but the genomic change recognized by most scholars is MYB-NFIB gene fusion.

Several studies have shown that AdCC carries the t (6; 9) (q22-23; p23-24) translocation, resulting in MYB-NFIB gene fusion, which may be the main pathogenic driver of AdCC.^[13,17,18]^ This finding was confirmed by MYB RNA overexpression, which was detected in both fusion-positive and -negative ACC.^[13,17]^ SB-AdCC, a TNBC, generally lacks the TP53 and PIK3CA mutations common in TNBC, while the incidence of MYB gene rearrangements is lower than in C-AdCC, resulting in generally lower MYB protein expression.^[1,19–23]^ However, MYB-NFIB gene fusion in salivary gland and breast AdCC appears to be unrelated to patient prognosis.^[24]^

The misdiagnosis rate of breast AdCC is high, reaching approximately 50%.^[6]^ To avoid overtreatment of AdCC and reduce iatrogenic harm to patients, it is particularly important to make a correct pathohistological diagnosis. C-AdCC should be differentiated from infiltrating cribriform carcinoma, cribriform ductal carcinoma in situ, adenoid infiltrating ductal carcinoma, and collagen corpuscle disease, while SB-AdCC should be differentiated from small cell carcinoma (neuroendocrine carcinoma), solid papillary carcinoma, metaplastic carcinoma, metastatic carcinoma, and rare malignant lymphoma.^[25]^

The 10-year survival rate of breast AdCC patients exceeds 90%.^[6,26,27]^ Ro et al suggested that the surgical method should be selected according to the histological grade. Local resection of tumors is indicated for grade I tumors, mastectomy for grade II tumors, and mastectomy plus lymph node dissection for grade III tumors.^[28]^

The incidence of distant metastasis of AdCC of the breast is <20%, with the lungs and bones as the most common destination organs. By contrast, TNBC has a high frequency of lymph node and distant metastases, with the brain and lungs as the most common destination organs.^[29,30]^

The axillary lymph node metastasis rate of AdCC is <8%.^[6,31–33]^ However, SB-AdCC has a higher incidence of axillary lymph node metastasis than C-AdCC, indicating a more aggressive clinical course.^[34,35]^ Axillary lymph node dissection is not necessary if there is no clear clinical evidence before surgery.^[25,36–38]^

Postoperative adjuvant radiotherapy may improve the overall- and progression-free survival of patients.^[26,39]^ However, there is still no consensus on this issue, as most studies have shown that postoperative adjuvant chemotherapy cannot improve the overall- and progression-free survival of patients.^[1,36,37]^ However, for patients with a tumor diameter >3 cm, axillary lymph node metastasis, and AdCC with high-grade transformation, adjuvant chemotherapy is necessary.^[38]^ AdCC is often considered a histological subtype of TNBC, implying that endocrine therapy is not unnecessary.^[10]^

## 4. Conclusions

Due to the high misdiagnosis rate of AdCC, accurate histopathological diagnosis is particularly important. At present, breast conserving surgery and local tumor resection are mainly used for the treatment of breast AdCC, and postoperative adjuvant radiotherapy is feasible. By contrast, the need for postoperative adjuvant chemotherapy, axillary lymph node dissection, and endocrine therapy should be determined based on the patient condition and comprehensive evaluation.

## Author contributions

**Conceptualization:** Wen Bin Gou, Pei He.

**Data curation:** Yong Qiang Yang, Bei Wen Song.

**Methodology:** Yong Qiang Yang, Bei Wen Song.

**Supervision:** Bei Wen Song.

**Writing – original draft:** Wen Bin Gou.

**Writing – review & editing:** Wen Bin Gou, Pei He.
